# Unraveling the shared genetic architecture of metabolic bone diseases via genomic structural equation modeling and multi-omics integration

**DOI:** 10.1371/journal.pone.0360386

**Published:** 2026-10-27

**Authors:** Kun Yi, Fan Wang, Feiping Hu, Wenlong Tang, Yang Liu, Bowen Gao, Sining Zhang, Lang Jiang, Jiaxin Ding, Daoping Lei, Junjie Yu, Siyuan Yu, Binghua Wu

**Affiliations:** Department of Orthopedics, Affiliated Rehabilitation Hospital of Nanchang University, Nanchang University, Nanchang, China; International Medical University, MALAYSIA

## Abstract

**Background:**

The shared genetic etiology underlying the comorbidity of skeletal degeneration and metabolic dysregulation remains poorly understood. This study aims to decipher the pleiotropic genetic architecture of multivariate metabolic bone diseases (mvMBDs) through an integrative summary-statistics framework.

**Methods:**

We applied Genomic Structural Equation Modeling (Genomic SEM) to integrate genome-wide association study (GWAS) summary statistics for six complex traits: osteoporosis (OP), chronic kidney disease (CKD), type 2 diabetes (T2D), alkaline phosphatase (ALP), high-density lipoprotein (HDL), and C-reactive protein (CRP). Subsequent analyses incorporated robust fine-mapping (SuSiE/FINEMAP), transcriptome-wide association studies (TWAS), and partitioned heritability assessments to identify putative functional loci and regulatory mechanisms.

**Results:**

Genomic SEM revealed significant genetic covariance among the six traits, successfully deriving a latent mvMBD construct. We identified 197 genomic risk loci, including 193 lead SNPs that did not achieve genome-wide significance in the six constituent univariate GWAS, predominantly localized in non-coding cis-regulatory elements (e.g., super-enhancers and intronic regions). Fine-mapping and TWAS prioritized critical pleiotropic genes (e.g., *MACF1*, *CETP*, *SPP1*, and *HNF1A*). Gene-set enrichment analyses identified enrichment for terms related to bone biology, lipid metabolism, and inflammation.

**Conclusion:**

Our findings robustly demonstrate that mvMBDs are not isolated clinical entities but rather interconnected systemic syndromes underpinned by glycolipid metabolic dysregulation and shared pleiotropic regulatory networks. This study establishes a novel theoretical paradigm for the genetic basis of metabolic bone diseases, offering promising functional targets for future precision medicine interventions.

## Introduction

As Hippocrates profoundly stated, “It is the business of the physician to know the nature of the body and the bone, for this is the beginning of all knowledge.” Metabolic bone diseases (MBDs) extend far beyond localized bone mass loss and microarchitectural deterioration; rather, they constitute a highly dynamic and cross-systemic biological process. This pathogenesis involves complex interactions among multi-organ metabolic networks and the progressive deterioration of a chronic osteoimmune inflammatory microenvironment [1]. Driven by an accelerating global aging population, the escalating incidence of MBDs has emerged as a significant medical and socioeconomic challenge [2]. Recently, the emergence of comprehensive biobanks and advances in high-throughput genotyping have profoundly accelerated the discovery of risk variants via genome-wide association studies (GWAS). Large-scale GWAS have yielded significant breakthroughs in identifying susceptibility loci for isolated skeletal phenotypes, greatly enhancing our understanding of bone pathology [3].

While conventional univariate GWAS are highly effective for estimating trait-specific associations, they do not directly model cross-trait covariance [4]. As a result, relying solely on single-phenotype definitions limits the ability to capture the intricate, systemic genetic networks underlying multifaceted syndromes like MBDs. Consequently, our comprehensive understanding of the pleiotropic genetic architecture and underlying biological mechanisms of MBDs remains largely unclear. Previous studies highlight specific signaling mutations and osteoblast-osteoclast uncoupling as key drivers of bone disease [5]. However, these localized mechanisms fail to capture the vast interindividual heterogeneity in systemic multimorbidities, such as the co-occurrence of diabetes mellitus or chronic kidney disease [6].

To overcome these methodological constraints and precisely address the limitations of univariate approaches, we employed Genomic Structural Equation Modeling (Genomic-SEM) to conduct a GWAS on a latent, unobserved composite phenotype [7]. First, it defines a unified latent factor by synthesizing the genetic covariance among multiple interconnected traits. These traits include osteoporosis (OP), systemic metabolic disorders such as type 2 diabetes (T2D) and chronic kidney disease (CKD), and circulating biomarkers such as C-reactive protein (CRP), high-density lipoprotein (HDL), and alkaline phosphatase (ALP). By synthesizing large-scale GWAS summary statistics across diverse MBD-related comorbidities and core clinical biomarkers, we constructed a novel, multifaceted latent construct, which we termed “mvMBDs” (multivariate Metabolic Bone Diseases). Integrating a systems biology perspective, we systematically interrogated the shared genetic variation, effectively stripped of phenotype-specific confounding noise, as the fundamental genetic architecture driving this complex pathological network [8]. Ultimately, this study dismantles the analytical barriers of traditional single-phenotype GWAS, revealing the shared genomic loci driving MBDs and providing a robust theoretical foundation for clinical intervention.

## Methods

### Research workflow and overview

To systematically investigate the shared genetic architecture of MBDs, our analytical pipeline comprised four primary modules (Fig 1). First, we compiled large-scale univariate GWAS summary statistics across six interconnected metabolic and skeletal traits. Second, we implemented Genomic SEM to estimate a common factor model across the six traits, deriving summary statistics for the multivariate phenotype (mvMBDs). Third, we characterized the genomic risk loci of mvMBDs through functional annotation, fine-mapping, and transcriptome-wide association studies (TWAS). Finally, we performed multilevel enrichment analyses spanning gene, disease ontology, and cell-type-specific levels.

**Fig 1:**
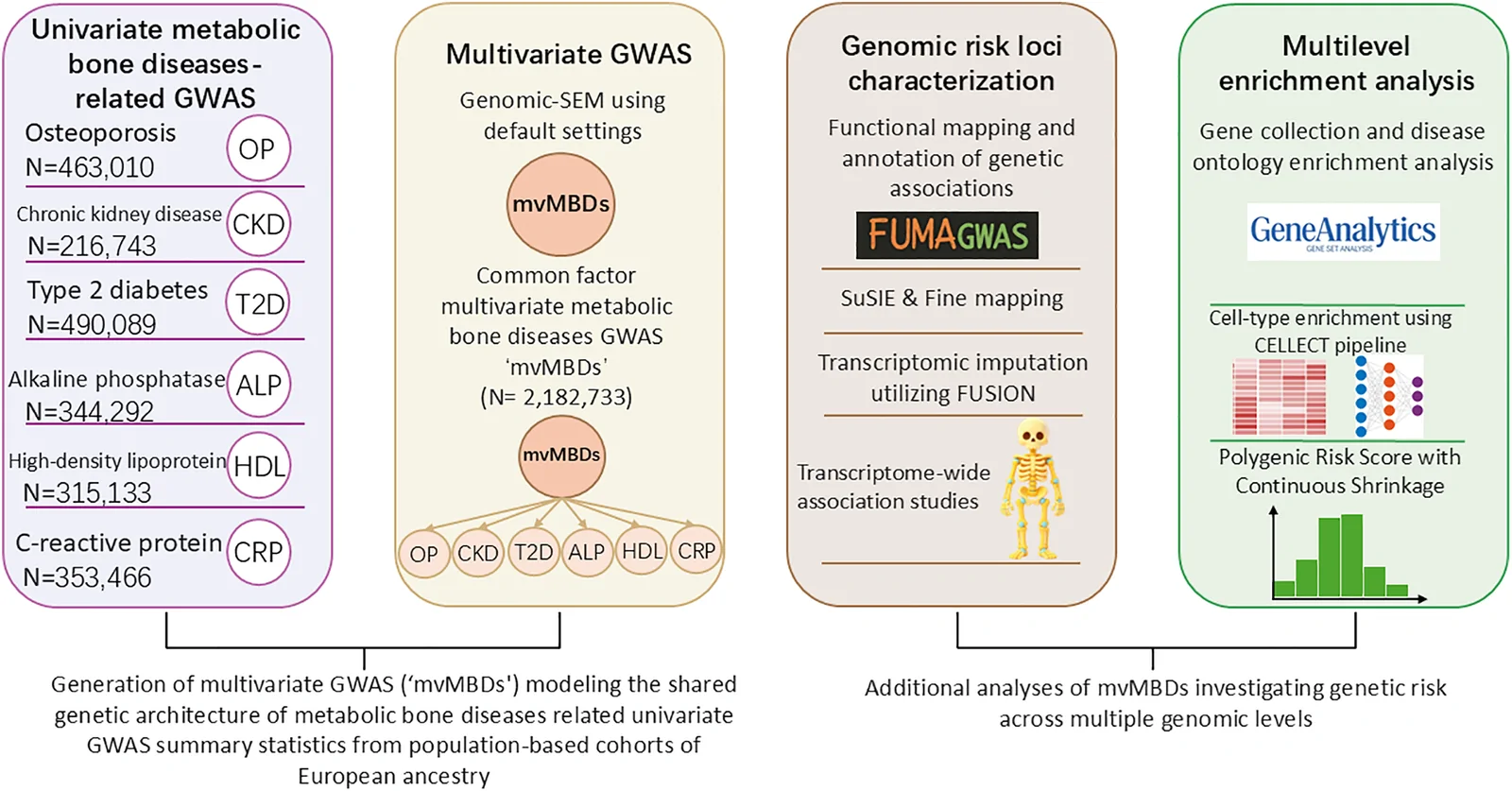
Research Workflow Diagram. This diagram summarizes the data sources, analytical pipeline, and methodologies employed in this study. Abbreviations: GWAS, Genome-wide association study; CELLECT, CELL-type-specific expression integration for complex traits.

### Phenotype selection and univariate GWAS data sources

The univariate GWAS summary statistics utilized to investigate MBDs were sourced from six distinct studies encompassing bone metabolism-related phenotypes: OP, CKD, T2D, ALP, HDL, and CRP. These six traits were purposefully selected to comprehensively capture the osteoimmune-metabolic axis, as T2D and CKD frequently disrupt bone microarchitecture, while ALP, HDL, and CRP serve as robust biomarkers for bone turnover, lipid metabolism, and chronic inflammation. All original cohorts contributing the inputted GWAS datasets received ethical approval from their respective Institutional Review Boards (IRBs) and obtained informed consent from all participants. Furthermore, all datasets underwent rigorous quality control (QC) procedures by their respective original consortia, such as filtering for imputation quality and minor allele frequency(MAF).

The GWAS summary statistics for OP, CKD, T2D, ALP, HDL, and CRP were sourced from publicly available datasets via the MRC IEU OpenGWAS infrastructure, encompassing large-scale cohorts from the UK Biobank (for OP) [9], the FinnGen consortium (for CKD) [10], and the multi-trait GWAS atlas (for T2D, ALP, HDL, and CRP) [11]. All included GWAS datasets were restricted to populations of European ancestry (EUR) to minimize potential biases arising from ancestral heterogeneity and population stratification. All GWAS summary statistics used in this study were accessed between January 2026 and March 2026. The authors did not have access to any information that could identify individual participants during or after data collection. Detailed descriptions of the GWAS datasets, including their IEU GWAS IDs, specific phenotypes, exact case-control counts, and total sample sizes, are systematically documented in Table 1.

**Table 1:** Single-Input GWAS.

IEU GWAS ID	Phenotype	Cases	Controls	Sample size	Ancestry
ukb-b-12141	OP	1976	461034	463010	European
finn-b-N14_CHRONKIDNEYDIS	CKD	3902	212841	216743	European
ebi-a-GCST90018926	T2D	38841	451248	490089	European
ebi-a-GCST90018942	ALP	NA	NA	344292	European
ebi-a-GCST90018956	HDL	NA	NA	315133	European
ebi-a-GCST90018950	CRP	NA	NA	353466	European

### Quality control for single-input GWAS

Prior to our study, individual-level quality control was performed by the original investigators of the inputted GWAS, where samples exhibiting a missingness rate exceeding 5% were strictly excluded. Furthermore, the major histocompatibility complex (MHC) region (chromosome 6, 25,000,000–35,000,000 bp, based on the GRCh37/hg19 human genome assembly) required specific handling. Due to its extensive genetic diversity and structural complexity,particularly the extreme polymorphism of immune-related genes,this region is known to severely confound linkage disequilibrium (LD) estimation [12]. In preparing the summary statistics for the mvMBDs construct, we explicitly defined our quality control pipeline. We retained all autosomal SNPs from the six inputted GWAS that satisfied strict filtering criteria. These genetic variants were subsequently harmonized against the 1000 Genomes Phase 3 European (EUR) reference panel [13]. To ensure the robustness of the downstream Genomic-SEM, stringent filtering criteria were implemented to remove: (i) SNPs with a minor allele frequency < 0.01, as rare variants are highly prone to genotyping errors due to small cluster sizes and typically yield inflated standard errors in LD score regression (LDSC) [7,12]; (ii) SNPs with an effect size estimate of exactly zero, which is imperative to prevent matrix non-invertibility issues during model fitting [7,12]; and (iii) SNPs presenting with mismatched alleles or strand ambiguities relative to the reference panel.

### Sample overlap in single-input GWAS

In our analysis, the incorporated GWAS datasets were acquired from distinct cohorts that share common participants (e.g., UK Biobank). The statistical dependencies introduced by potential sample overlap and shared population stratification across these cohorts were explicitly estimated and adjusted for using the multivariable linkage disequilibrium score regression (LDSC) sampling covariance matrix [12,14]. The cross-trait LDSC intercepts inherently capture and correct for inflated phenotypic correlations arising from participant overlap without requiring individual-level overlap proportions to be pre-specified, thereby safeguarding against confounding in downstream multivariable modeling.

### Genomic structural equation modeling

To investigate the pervasive genetic liability underlying these multivariate metabolic bone disease-related traits, we conducted Genomic-SEM on OP, CKD, T2D, ALP, HDL, and CRP using the GenomicSEM R package (v.0.0.5) [7]. Genomic SEM is an advanced methodological framework that models the joint genetic architecture of complex traits by synthesizing empirical genetic covariance matrices [7]. Detailed LDSC diagnostics for the six input GWAS are provided in Table 2.

**Table 2:** LDSC diagnostics for the six input GWAS.

Phenotype	NSNPs	h2	λGC	Mean_χ2	Intercept	Ratio
OP	1081323	0.0127	1.0966	1.1478	1.0271	0.1833
CKD	1153045	0.0025	1.0426	1.0389	1.0282	0.7266
T2D	1171063	0.0608	1.4062	1.6652	1.0904	0.136
ALP	1160193	0.1566	1.5903	2.4111	1.2314	0.164
HDL	1160975	0.1823	1.7688	2.3919	1.2265	0.1628
CRP	1161387	0.125	1.6305	2.0241	1.1275	0.1245

The Genomic SEM framework is executed in two distinct stages. In the first stage, the empirical genetic covariance matrix and its corresponding sampling covariance matrix are estimated [7]. We prepared the summary statistics of the inputted GWAS and utilized a multivariate extension of cross-trait LDSC to generate the empirical genetic covariance matrix among the six traits, which served as the foundation for the subsequent common factor SEM (S1 File) [14]. The second stage involves specifying an SEM model optimized to minimize the discrepancy between the model-implied (hypothesized) covariance matrix and the empirically derived covariance matrix calculated in the first stage. Given our primary research objective to delineate the shared genetic liability underlying the MBD-related traits, we specified and evaluated a common single-factor model. Model fit was evaluated using standardized metrics, including the standardized root mean square residual (SRMR), model χ2, Akaike Information Criterion (AIC), and Comparative Fit Index (CFI) (S1 Table) [7,15]. Finally, by embedding individual autosomal SNP associations into the established genetic and sampling covariance matrices under this validated single-factor specification, we generated comprehensive multivariate GWAS summary statistics for 4,911,367 SNPs, effectively capturing the shared genetic covariance underlying the mvMBDs network.

### SNP heterogeneity in genomic SEM

To evaluate whether SNP associations conformed to the common factor specification within the Genomic SEM framework, we calculated the SNP heterogeneity statistic QSNP [7]. The null hypothesis for this test posits that the effect of a given SNP on the constituent univariate GWAS phenotypes is fully mediated by the latent common factor. A statistically significant QSNP test indicates heterogeneity relative to the factor model, reflecting trait-specific or residual associations not accounted for by the shared factor construct.

### Evaluation of significance thresholds and LDSC parameters

To evaluate the stability of multivariate GWAS signals across varying stringency thresholds, we examined locus yields at standard genome-wide significance (p < 5 × 10^-8^) as well as more stringent thresholds (p < 5 × 10^-12^ and p < 5 × 10^-16^). In addition, LDSC parameters and sampling covariance matrices were estimated following the standard two-step LDSC protocol [12]. Specifically, to prevent large-effect association peaks from upwardly biasing the regression intercept, the two-step estimator fitted the regression intercept using variants with chi-square values < 30 before estimating the heritability and covariance slopes across all quality-controlled SNPs [12].

### Definition of genomic loci and locus comparison

We utilized the Functional Mapping and Annotation of Genome-Wide Association Studies (FUMA) platform to define genomic risk loci and identify independent lead SNPs associated with mvMBDs [16]. These lead SNPs were rigorously defined based on a stringent LD threshold (r^2^ < 0.1) and a genome-wide significance threshold (p < 5 × 10^−8^). Initially, the summary statistics of the mvMBDs GWAS were uploaded to FUMA to evaluate the strength of the genetic associations. We further compared the identified lead SNP loci with those from the original constituent univariate GWAS. To determine whether the 70 identified lead SNPs for mvMBDs exhibited pleiotropic associations, we systematically queried the NHGRI-EBI GWAS Catalog for previously published significant associations (p < 5 × 10^−8^) [17].Furthermore, exploiting the integrated Multi-marker Analysis of GenoMic Annotation (MAGMA) framework within FUMA, we conducted a gene-level analysis for the established mvMBDs model [18]. As a robust post-GWAS analytical tool, MAGMA aggregates multiple genetic markers (e.g., SNPs) into a single gene-level signal, extracting functional genetic information from genome-wide summary statistics to rigorously evaluate gene-phenotype associations. The significance threshold for this gene-level analysis was strictly set at a false discovery rate (FDR) <0.05. Additionally, we implemented a subtraction filtering procedure. Briefly, this procedure involves cross-referencing the lead loci identified via Genomic SEM against the genome-wide significant loci derived from each constituent univariate GWAS. By filtering out loci that were already genome-wide significant in the individual input traits, this approach isolates susceptibility loci specifically revealed by the multivariate latent construct.

### Fine-mapping

To prioritize the most probable putative candidate variants underlying the mvMBDs associations, we conducted rigorous statistical fine-mapping using the Sum of Single Effects (SuSiE) [19] and FINEMAP [20] algorithms, both implemented seamlessly within the echolocatoR R package (v.2.0.3) [21]. These state-of-the-art probabilistic frameworks are specifically designed to disentangle complex LD patterns and prioritize putative candidate variants. For this procedure, we defined a 250-kb genomic window flanking each identified lead SNP to compute the posterior inclusion probability (PIP) for all variants within the target loci. For each locus, a 95% credible set was constructed by ranking variants in descending order of their PIPs and selecting the minimal set of variants whose cumulative posterior probability reached at least 0.95 (∑PIP ≥ 0.95). Furthermore, individual PIP values were used to quantify variant-level causal evidence, and high-confidence consensus variants nominated across both SuSiE and FINEMAP were prioritized using the echolocatoR pipeline.

### Transcriptome-wide association study (TWAS)

Following the localization of putative candidate variants, we conducted a TWAS to prioritize candidate genes associated with mvMBDs by leveraging predicted gene expression-phenotype relationships [22]. This TWAS was executed using the FUSION pipeline, integrating 37,920 pre-computed expression quantitative trait loci (eQTL) weight profiles (representing specific gene-tissue pairs) derived from the Genotype-Tissue Expression (GTEx) v8 reference panel [23]. These reference weights facilitated the imputation of tissue-specific gene expression levels. For the downstream analysis, the inputted mvMBDs summary statistics contained sufficient overlapping variants to successfully evaluate 36,149 of the 37,920 eQTL features, thereby demonstrating robust data quality and high imputation coverage. Transcriptome-wide significance was defined using Bonferroni correction adjusting for the total number of evaluated eQTL features (p < 0.05 / 36,149 = p < 1.38 × 10^−6^), and significant genes were prioritized for downstream fine-mapping.

To refine these TWAS-significant signals, we applied the Fine-mapping Of CaUsal gene Sets (FOCUS) methodology, a probabilistic framework specifically engineered to fine-map TWAS associations [24]. The FOCUS pipeline was employed to prioritize candidate mvMBDs genes by calculating a PIP for each gene, which quantifies the posterior probability that the gene explains the observed TWAS signal. Genes that achieved TWAS significance and exhibited high posterior inclusion probabilities in FOCUS were prioritized as high-confidence candidate genes associated with mvMBDs.

### Gene set and disease ontology enrichment analysis

To elucidate the potential biological interplay between mvMBDs and Mendelian disease genes, as well as their associated signaling pathways, we performed comprehensive gene-set and pathway enrichment analyses. These evaluations were executed by leveraging the MAGMA framework and Gene Set Enrichment Analysis (GSEA) integrated within the FUMA platform.

### Cell-type annotation analysis

To elucidate the etiological cell types driving the pathogenesis of mvMBDs, we implemented the Cell-type Expression-specific integration for Complex Traits (CELLECT) framework to analyze single-cell RNA sequencing (scRNA-seq) data [25]. Specifically, we leveraged the Tabula Muris dataset, a comprehensive transcriptomic atlas comprising over 100,000 individual cells spanning 20 distinct organs and tissues from the mouse (Mus musculus) [26]. To accurately quantify cell-type-specific gene expression patterns, the Tabula Muris scRNA-seq data were rigorously preprocessed and normalized using the CELLEX (Cell-Type Expression Specificity) algorithm, which computes a highly robust expression specificity probability score for each gene [25]. Subsequently, stratified LDSC was invoked to integrate these cell-type specificity scores with our multivariate GWAS summary statistics, thereby facilitating the evaluation of cell-type-specific genetic heritability enrichment [27]. Significant cellular etiologies were systematically categorized and identified by applying a stringent FDR threshold of < 0.05.

### Partitioned heritability analysis across functional genomic categories

To quantify the proportion of phenotypic variance explained by specific functional categories, we performed partitioned heritability analysis. This analytical approach systematically partitions the genetic architecture of the target phenotypes across diverse functional genomic annotations (e.g., protein-coding genes, active enhancers, and repressors) to evaluate the relative contribution of each genomic region to the overall SNP-based heritability. Specifically, stratified LDSC was employed to integrate pre-computed weighted LD matrices, allele frequency profiles, and our multivariate summary statistics. This statistical framework rigorously computes heritability enrichment adjusting for linkage disequilibrium. Specifically, we utilized the baseline v1.1 model, partitioning the genetic architecture across 53 distinct functional genomic categories (including coding regions, conserved regions, enhancers, and histone marks) to establish a robust genomic baseline [28].

## Results

### Statistical metrics for structural equation modeling (SEM)

Initial multivariable LDSC analyses revealed robust genetic signals for the inputted GWAS traits. Specifically, the SNP-based heritability Z-scores (h^2^ Z) for OP, CKD, T2D, ALP, HDL, and CRP were 8.25, 1.26, 13.8, 10.7, 16.7, and 12.6, respectively. Within the Genomic SEM framework, an h^2^ Z > 4 is conventionally recommended as an empirical heuristic to ensure a sufficient signal-to-noise ratio for stable downstream multivariable modeling. Notably, while the h^2^ Z for CKD (h^2^ Z = 1.26) was comparatively modest, it was intentionally retained in the joint model given its clinical and biological relevance to the renal-bone axis, and its inclusion did not compromise the positive definiteness of the genetic covariance matrix. Furthermore, cross-trait LDSC demonstrated pervasive genetic correlations among these diverse phenotypes (detailed single-factor genetic parameters are provided in S2 Table; Figs 2A-2B) [3]. The common factor model yielded a robust fit when aligning the model-implied genetic covariance matrix of the six inputted GWAS with the empirically derived covariance matrix, quantitatively supported by excellent goodness-of-fit indices (CFI = 0.956, SRMR = 0.06) (comprehensive evaluations of model stability, along with parameters for the latent factor [F1] and univariate SEMs, are detailed in S1 Table) [7]. Standardized factor loadings indicated that the latent construct is primarily driven by systemic metabolic and inflammatory components (HDL, CRP, CKD, and T2D), with comparatively modest loadings observed for isolated skeletal phenotypes (OP and ALP) (Fig 2B). Collectively, these findings provide compelling statistical evidence for a shared latent genetic architecture driving mvMBDs. Moreover, the multivariate genome-wide association analysis of the derived latent mvMBDs construct exhibited multiple distinct association peaks that substantially surpassed the stringent genome-wide significance threshold (p < 5 × 10^−8^) (Fig 2C), offering direct visual confirmation of the highly polygenic nature inherent to MBDs.

**Fig 2:**
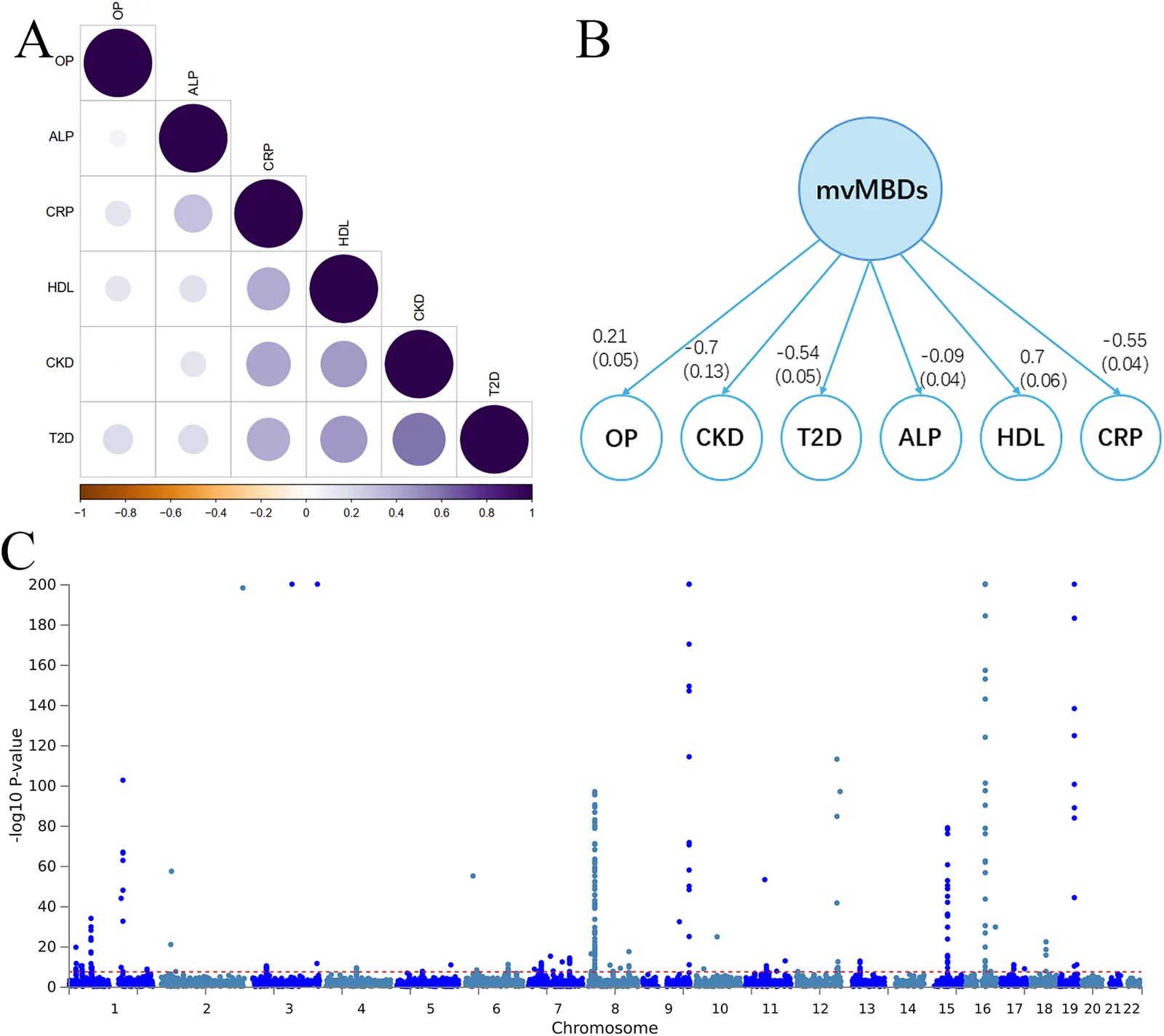
Multivariate MBDs GWAS using genomic-SEM. (A) Genetic correlations estimated via LD scores for six single-trait GWAS, the color gradient and size of the circles represent the magnitude and direction of the genetic correlations (*r*_*g*_), with the color bar ranging from −1 (orange) to 1 (dark purple). (B) Heritability contributions of traits with standardized factor loadings (standard deviations in parentheses), the direction (sign) of the standardized factor loadings accurately reflects the underlying genetic correlations among the constituent traits; traits with loadings of the same sign are positively genetically correlated, whereas those with opposite signs are inversely correlated. (C) Manhattan plot of SNP associations (-log10(p-value)) across chromosomes.

### Hierarchical genomic-SEM GWAS evaluation

By applying the Genomic SEM framework to model the shared genetic covariance across these traits, we generated an indirectly measured GWAS for the unobserved latent construct, estimating the associations of 4,911,367 SNPs with mvMBDs (detailed in the Zenodo repository, https://doi.org/10.5281/zenodo.20457329).Applying a stringent threshold of p < 5 × 10^-12^, we identified a total of 197 genomic risk loci, encompassing 156 highly significant genome-wide loci (p < 5 × 10^-16^; S3 Table). This tiered screening strategy was intentionally implemented to comprehensively capture reliable signals while preventing the omission of biologically meaningful loci. Across varying significance thresholds, 193 of these 197 lead SNPs were not genome-wide significant in the six constituent univariate GWAS used to construct mvMBDs (S4 Table), highlighting loci captured through joint multivariate modeling. This underscores the substantially enhanced discovery power afforded by the Genomic SEM approach [29]. These identified mvMBD lead SNPs were predominantly enriched for traits fundamentally linked to metabolic syndrome (MetS), lipid profiles, CRP, renal function, and T2D. Among the highly significant SNPs (p < 5 × 10^-16^), only four lead SNPs had been previously reported in the existing literature (comprehensive identification reports are provided in S5 Table). Notably, specific variants, including rs115849089, rs72786786, and rs11635491, demonstrated established pleiotropic associations with mvMBD-related traits [30]. Ultimately, by applying Genomic SEM, this study identified mvMBD-associated SNPs across multiple levels of genetic correlation. Furthermore, these identified loci are associated with biological pathways related to lipid metabolism, systemic inflammation, and metabolic multimorbidity [1].

### Genomic-SEM control via LD score regression

From the initial pool of 4,911,367 SNPs, stringent parameter controls and regression coefficient filtering resulted in the retention of 1,003,804 valid SNPs for downstream evaluation. Assessment of the summary statistics yielded a mean χ2 statistic of 1.224 and a genomic control inflation factor λGC of 1.128, alongside a maximum χ2 value of 913.763. Furthermore, we identified 126 genome-wide significant loci, and the heterogeneity test confirmed the absence of significant pleiotropic heterogeneity (p > 0.05). Crucially, the LDSC regression model yielded an intercept of 0.9936 ± 0.0092 (standard error) and a ratio of < 0 [12]. Collectively, these parameters indicate that the observed statistical inflation within our multivariate genome-wide association analysis is primarily driven by a true polygenic signal, rather than being substantially confounded by population stratification biases or cryptic pleiotropy.

### FUMA-based evaluation of mvMBDs SEM

Subsequent evaluation of the Genomic SEM summary statistics via the FUMA pipeline identified a total of 59 independent genomic risk loci (S6 Table, Fig 3, the individual regional association plots for each locus are compiled in S2 File.7z). Furthermore, robust multiple-testing correction (genome-wide significance p < 5 × 10^−8^, FDR < 0.05) prioritized 148 putative mvMBD-associated genes. Functional annotation driven by FUMA delineated 70 distinct lead SNPs, the vast majority of which were localized to intergenic regions (S7 Table, Fig 4C). Furthermore, the application of this subtraction filtering approach isolated several key candidate variants, most notably rs10280461 and rs9594738 (S8 Table). Biologically, rs9594738 is proximally linked to RANKL (TNFSF11), a master cytokine driving osteoclastogenesis and bone resorption [31]. Conversely, rs10280461 maps to SFRP4, a critical signaling molecule that exerts broad pleiotropic control over both osteoblast-mediated bone formation and systemic glycolipid metabolism [32,33]. The identification of these specific loci profoundly corroborates the comorbid phenotypic architecture of structural bone loss and metabolic dysregulation investigated in this study. Finally, tissue-specific enrichment analyses did not identify any specific tissue type with statistically significant overrepresentation following multiple-testing correction (Fig 4D-4E). We acknowledge that this lack of significant tissue enrichment cannot be interpreted as evidence of a systemic syndrome; rather, this non-significant result may reflect limited statistical power, the use of broad reference annotations, underlying measurement noise, or the stringent multiple-testing burden.

**Fig 3:**
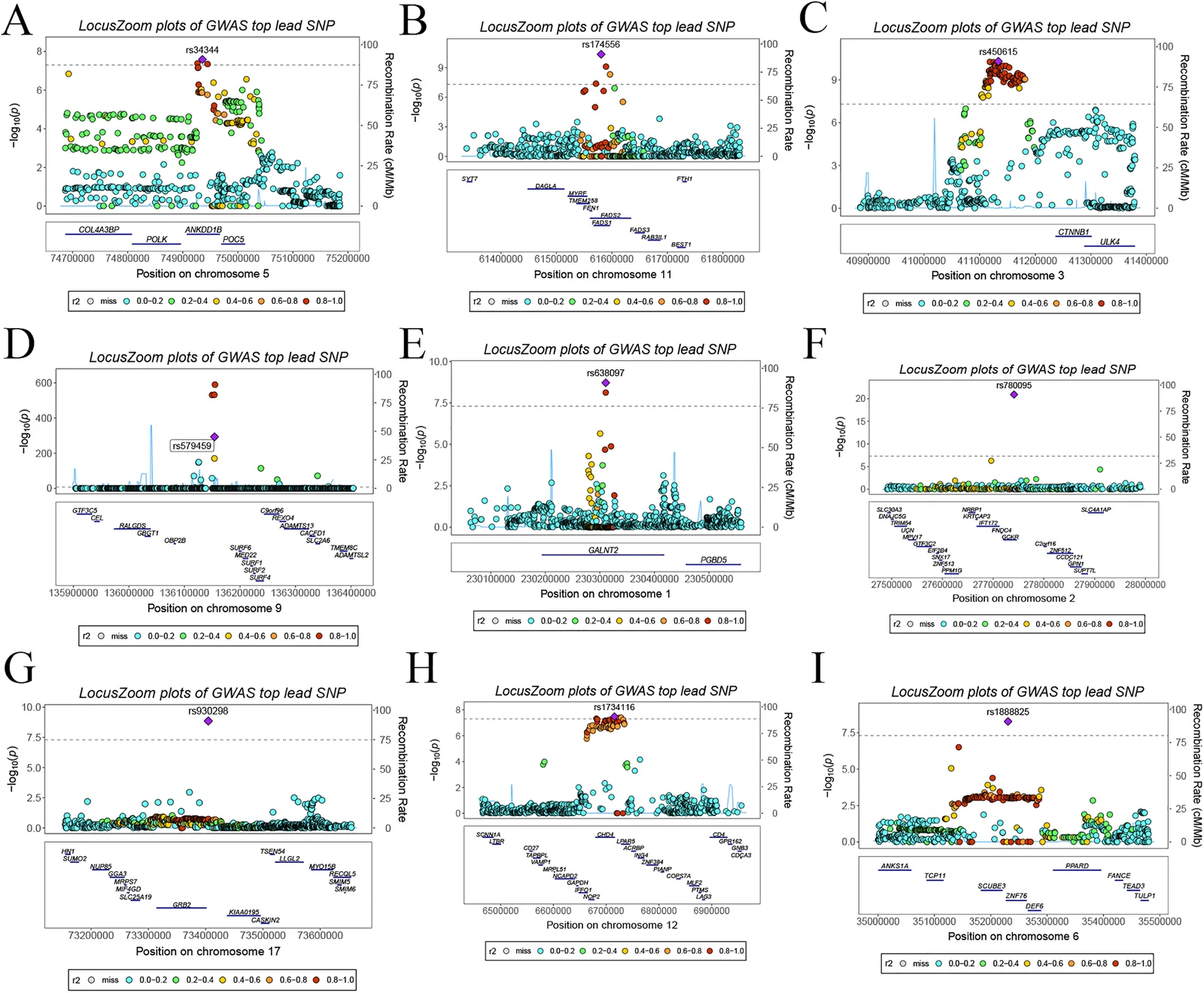
Details of mvMBDs risk loci. (A–I) Regional association plots for mvMBDs -related traits.

**Fig 4:**
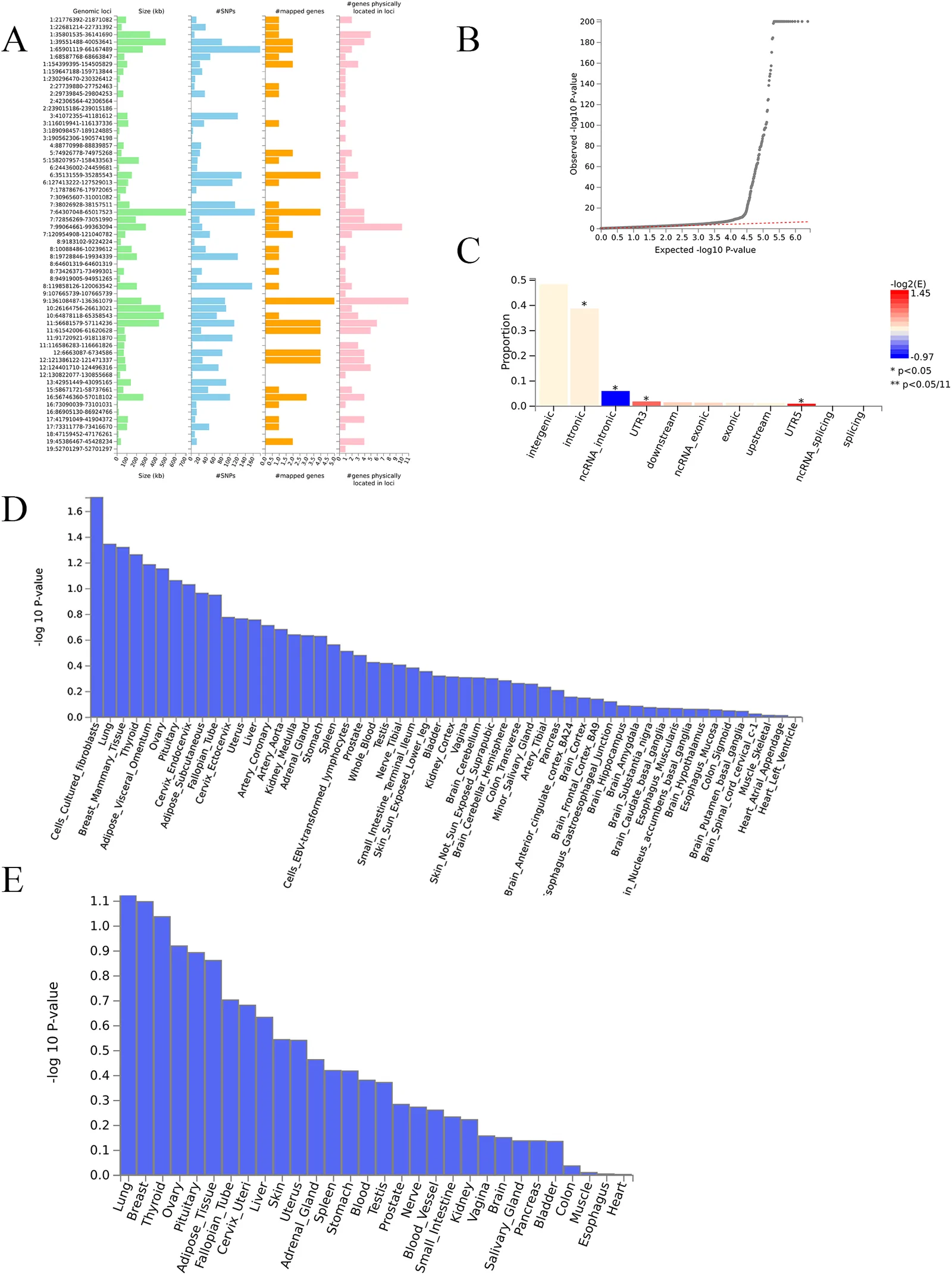
FUMA-based SEM evaluation. (A) mvMBDs genetic risk loci. (B) SEM goodness-of-fit assessment. (C) Regional annotation of lead SNPs. (D–E) SNP cell/tissue correlation rankings.

### Fine-mapping

Statistical fine-mapping analysis prioritized candidate variants underlying mvMBD associations across multiple distinct genomic loci. Specifically, robust association signals were pinpointed at chromosome 1 (rs61779330), chromosome 8 (rs2929470), chromosome 12 (rs113447364 and rs73226292), and chromosome 19 (rs72511605). Regional association plots visually corroborated these findings, illustrating prominent, sharply defined association peaks at these target loci [34]. Furthermore, substantial evidence of association was also observed among other linked variants comprising the 95% credible sets, collectively reinforcing the putative functional potential of these complex genomic regions (S9 Table, Fig 5A-5D) [35].

**Fig 5:**
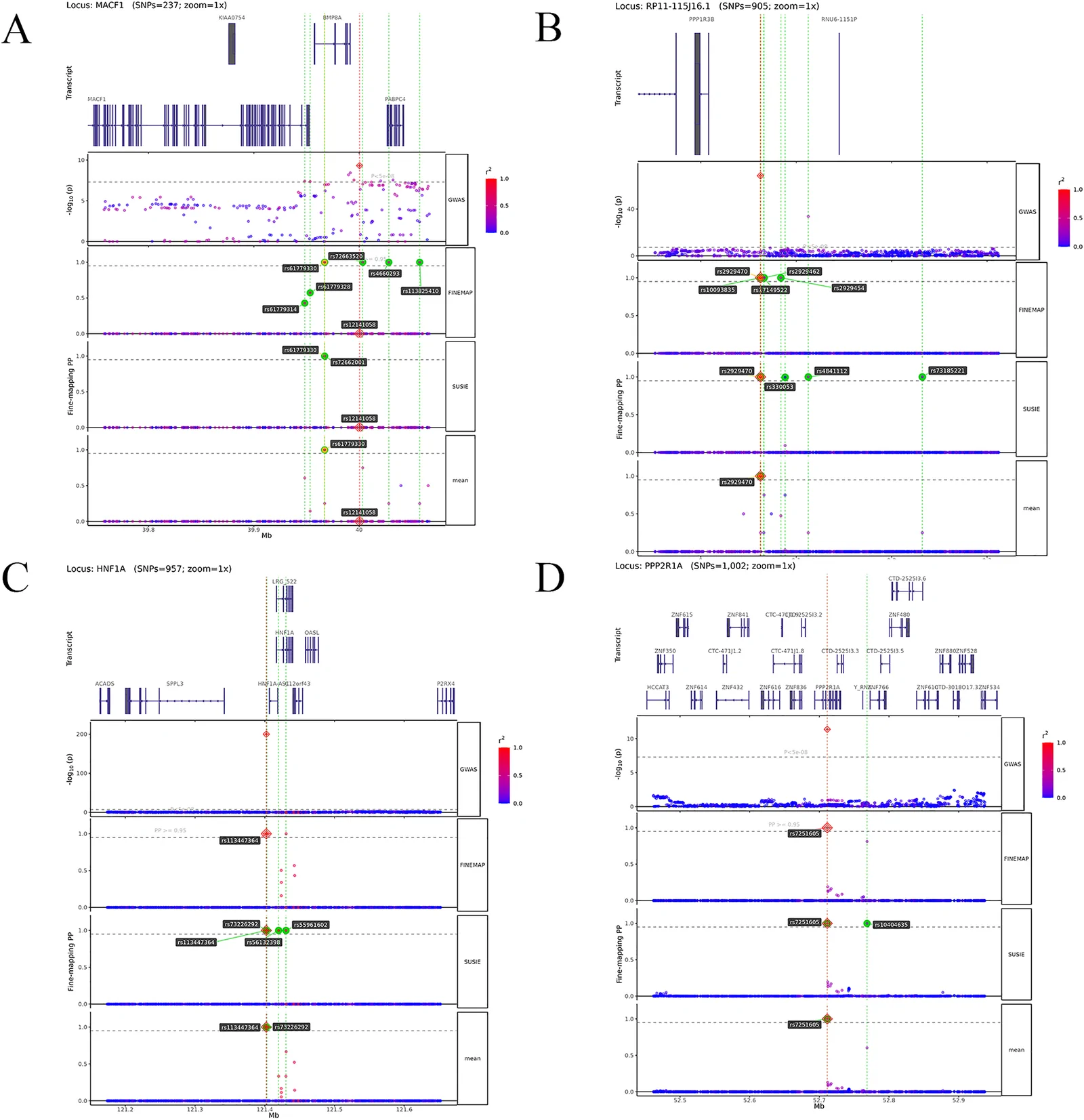
Some details of the mvMBDs Fine-mapping. A Fine-mapping results of rs61779330. B Fine-mapping results of rs2929470. C Fine-mapping results of rs113447364, rs73226292. D Fine-mapping results of rs72511605.

### Transcriptome-wide association predictions

To identify gene-level associations underlying the genetic architecture of mvMBDs, we subsequently performed a transcriptome-wide association study (TWAS) using the FUSION pipeline. This analysis revealed 12 genes that surpassed the transcriptome-wide Bonferroni-corrected significance threshold (p < 1.38 × 10^−6^ across 36,149 tested features; S10 Table). Following this, we subjected the Genomic SEM data to probabilistic fine-mapping utilizing the FOCUS framework, which prioritized 32 genes as high-confidence candidate signals for mvMBDs.

To robustly evaluate these gene-level associations, we performed an integrative analysis combining the TWAS and FOCUS results [24]. This yielded a consensus set of prioritized candidate genes: *CTC-366B18.4*, *LIN7C*, *C1R*, *CHKA*, *NLRC5*, *CPNE2*, *NFKBIB*, *CTC-487M23.5*, *BDNF-AS*, *RASGRP2*, *CKAP5*, *IFFO1*, and *ACRBP*. Among these, *CTC-366B18.4*, *LIN7C*, *C1R*, *CHKA*, *NLRC5*, *CPNE2*, and *NFKBIB* exhibited positive TWAS Z-scores (Z > 0), indicating that higher genetically predicted expression of these genes is associated with increased genetic liability to mvMBDs [36]. Conversely, *CTC-487M23.5*, *BDNF-AS*, *RASGRP2*, *CKAP5*, *IFFO1*, and *ACRBP* demonstrated negative TWAS Z-scores (Z < 0), indicating that lower genetically predicted expression of these genes is associated with increased disease liability (detailed results are provided in S11 Table, Fig 6).

**Fig 6:**
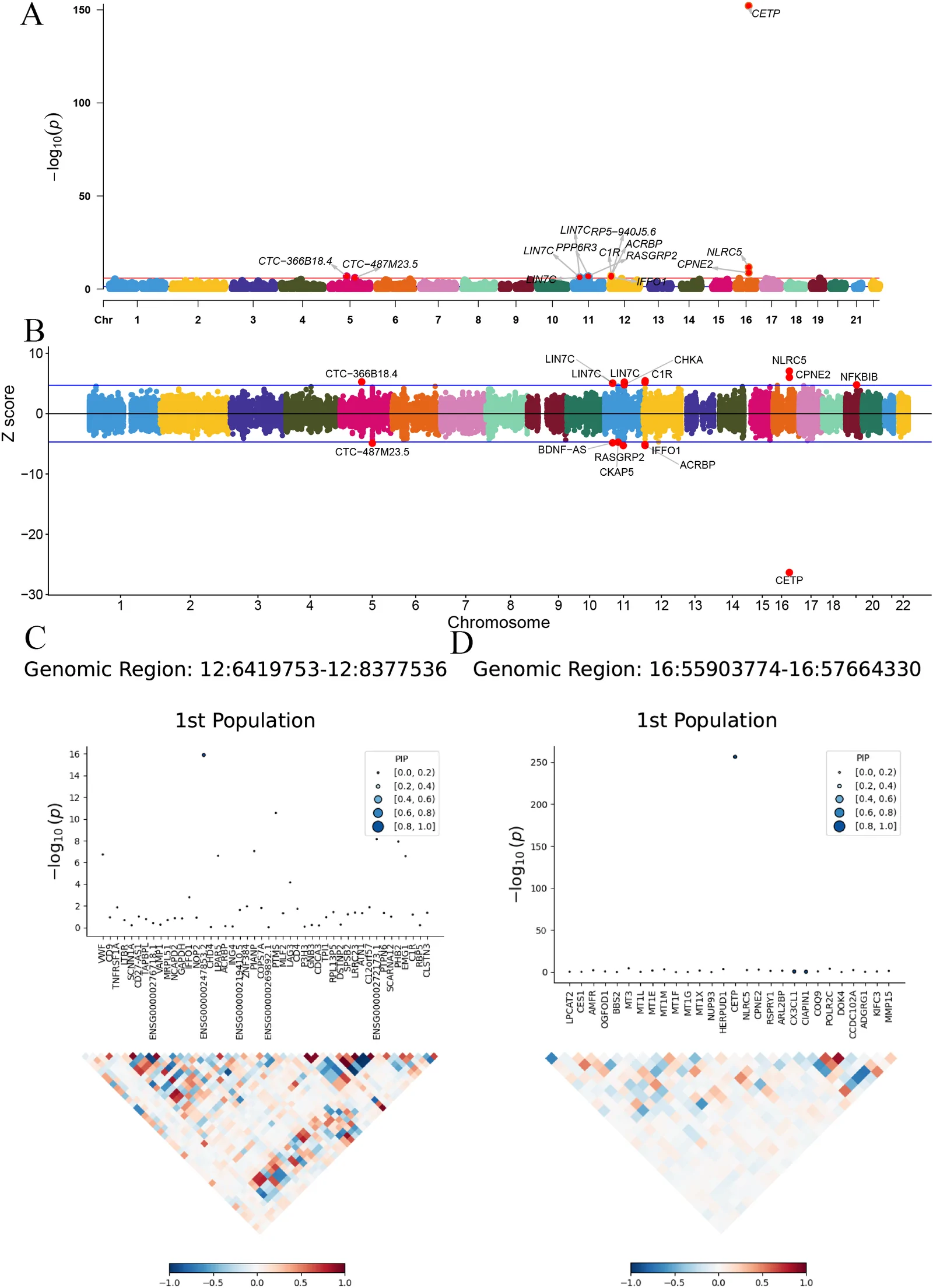
Transcriptome-wide association analysis using FUSION. A. Regional association plot of significant gene loci. B. Regional Z-score evaluation of significant gene loci. C. FOCUS-mapped significant gene locus ACRBP. D. FOCUS-mapped significant gene locus CETP.

### Pathway, cell-type, and mendelian disease enrichment

Multi-marker Analysis of GenoMic Annotation (MAGMA) gene mapping identified 27 distinct candidate genes (S12 Table) [18]. Utilizing these core genes, we conducted comprehensive gene-set analyses, which revealed highly significant, cross-system pleiotropic enrichment across multiple Gene Set Enrichment Analysis (GSEA) terms (S13 Table).

Notably, numerous gene sets were not only inextricably linked to lipid homeostasis and cardiometabolic dysregulation (e.g., abnormal triglyceride levels, altered high-/low-density lipoprotein cholesterol, metabolic syndrome, type 2 diabetes, and coronary heart disease) as well as neurodegenerative pathologies (e.g., Alzheimer’s disease and cognitive decline) [1], but were also robustly enriched in a diverse array of bone-related pathological and structural phenotypes (e.g., childhood bone mineral density [BMD], lumbar spine BMD, femoral neck BMD, heel BMD, and fracture risk).

Furthermore, cell-type-specific enrichment analyses evaluating diverse single-cell profiles identified two specific cellular etiologies that successfully surpassed the significance threshold following robust multiple-testing correction (p < 0.05): pancreatic A cells and tracheal mesenchymal cells (S14 Table) [27].

### Partitioned heritability across genomic regions

By conducting functional partitioning of the multivariate genetic architecture, we discovered that the majority of the disease-associated genetic liability was not uniformly distributed across the genome; rather, it was significantly enriched within non-coding genomic regulatory elements [28]. Specifically, the heritability contributions were predominantly concentrated in regions harboring histone modifications indicative of active chromatin states (most notably, H3K27ac-marked super-enhancers and H3K4me3-marked promoters) as well as crucial transcription factor binding sites (TFBS) [37]. These active cis-regulatory networks play a pivotal role in mediating gene expression and orchestrating subsequent physiological functions.Concurrently, evolutionarily conserved sequences and actively transcribed regions (including untranslated regions, UTRs) also demonstrated substantial heritability contributions. Collectively, these findings strongly suggest that the pathogenesis of this complex comorbid phenotype is not primarily attributable to protein-altering coding mutations, but is instead profoundly dependent upon the systemic remodeling of the underlying genomic regulatory landscape (S15 Table) [38].

## Discussion

In the present study, we comprehensively interrogated the genetic architecture underlying OP, CKD, T2D, ALP, HDL, and CRP by deploying an integrative summary-statistics analytical framework. This approach encompassed Genomic SEM, statistical fine-mapping, and transcriptome-wide association studies (TWAS). Through the joint multivariate synthesis of these complex traits, we delineated multiple genetic susceptibility markers underlying this shared disease construct. Crucially, our findings elucidate that the underlying genetic liability not only dictates the pathogenesis of mvMBDs, but also exerts a profound, life-course impact on individuals via intricate manifestations across gene regulatory networks, systemic risk factors, and specific cellular etiologies. Ultimately, this investigation establishes a robust theoretical paradigm for understanding how pleiotropic genetic loci sculpt the phenotypic landscape of mvMBDs, thereby yielding invaluable insights to inform the future implementation of precision medicine strategies and targeted public health interventions.

To further delineate this shared genetic architecture, our investigation elucidated the profound genetic covariance underlying OP, CKD, T2D, ALP, HDL, and CRP through Genomic SEM. The findings demonstrate that these distinct phenotypes share a common latent genetic architecture, with the derived multivariate metabolic bone disease (mvMBDs) construct exerting a particularly pronounced influence on ALP and CRP. This observation aligns seamlessly with the theoretical paradigm proposed by Karsenty et al. (2012) [1], suggesting that the pleiotropic impact of mvMBDs extends substantially beyond the classical pathological boundaries of OP, CKD, and T2D. Furthermore, the SEM framework conclusively corroborated the intricate genetic interplay among these six traits, implying that they do not manifest as isolated clinical entities; rather, they are inextricably intertwined, operating synergistically within a highly complex systemic network.

To elucidate the specific genetic contributors within this network, the present study, through Genomic SEM and subsequent fine-mapping analyses, identified multiple critical pleiotropic SNPs associated with systemic metabolic and skeletal degenerative traits, notably OP, T2D, and CKD. Functional annotation demonstrated that these identified SNPs are predominantly localized within intronic and intergenic regions, underscoring the central role of non-coding regions in driving the genetic etiology of this multi-trait disease network. This observation is consistent with previous large-scale genomic findings by Maurano et al. (2012) [38], which suggested the regulatory roles of non-coding variants; rather, they predominantly function as distal cis-regulatory elements,such as enhancers,to modulate the transcriptional expression of target genes, thereby orchestrating the development of complex polygenic traits [38]. Ultimately, these identified SNPs are closely associated with the core phenotypes characterizing MBDs, including glycolipid metabolic dysregulation, renal functional decline, and the deterioration of bone microarchitecture. Consequently, our findings provide candidate targets for future functional validation and offer valuable insights into the shared genetic architecture underlying mvMBDs. Building upon this broad architectural perspective to identify specific functional elements, the current study employed multiple robust fine-mapping algorithms (including SuSiE and FINEMAP) to successfully pinpoint several critical putative candidate SNPs within this genetic network (e.g., rs61779330, rs1346864, and rs113447364, which exhibited consensus PIPs approaching 1). These high-confidence variants precisely map to core genomic regions (such as *MACF1*, *WLS*, and *HNF1A*) that are closely associated with skeletal degeneration and endocrine metabolism.This finding is highly concordant with the landmark genome-wide investigation by Estrada et al. (2012) [39], which established that key loci governing bone mineral density (BMD) are typically driven by specific, strong-effect functional SNPs [39].

Through the precise resolution of these fine-mapped variants, we not only replicate previous associations but also deeply elucidate the underlying molecular mechanisms. For instance, classical targets like *MACF1* and *WLS* have been extensively validated for their profound involvement in core skeletal biological processes, including the osteogenic differentiation of mesenchymal stem cells (MSCs), the regulation of the Wnt signaling pathway, and endochondral ossification [40]. Concurrently, *HNF1A* is a well-recognized pleiotropic gene orchestrating systemic glycolipid metabolism, particularly in the pathogenesis of type 2 diabetes (T2D) [41]. Crucially, the robust capture of these genetic markers,which dually regulate bone microarchitecture and systemic metabolism,within our model provides compelling evidence that these genomic regions do not merely precipitate isolated bone loss or reduced BMD. Rather, they function as critical regulatory hubs, playing a decisive role in shaping the complex, systemic pleiotropic phenotype of mvMBDs.

To elucidate the underlying biological pathways and target genes through which these regulatory hubs exert their effects, we applied the FUSION TWAS pipeline to further prioritize putative functional target genes significantly associated with these pleiotropic SNPs. These genes are not only deeply integrated into core biological pathways,such as systemic lipid metabolism, bone matrix remodeling, and cellular coupling communication,but are also intricately intertwined with the complex pathological mechanisms of MBDs, including OP and osteoarthritis (OA). For instance, functional variants within critical targets of the systemic lipid metabolism pathway (e.g., *CETP*, which mediates cholesterol transport) may indirectly compromise bone quality by disrupting lipid homeostasis [42]. Conversely, the aberrant expression of matrix regulatory genes within the bone microenvironment (e.g., *SPP1*, encoding osteopontin) directly disrupts the delicate balance between bone matrix mineralization and osteoclast anchorage-dependent resorption, thereby profoundly increasing individual susceptibility to bone loss and fragility lesions.

Furthermore, genes pivotal to the osteoimmunology microenvironment and intercellular communication networks were prominently corroborated in the present study. Notably, we observed significant enrichment of chemokines intimately linked to osteoclast recruitment and inflammatory osteolysis (e.g., *CX3CL1*), receptors mediating bidirectional osteoblast-osteoclast crosstalk (e.g., *EPHB2*), and core factors governing immune-inflammatory responses (e.g., *NLRC5* and *DUSP3*) [43]. These findings provide molecular clues for understanding the interplay among lipid metabolism, the immune system, and bone homeostasis, while highlighting potential therapeutic targets underlying the progression of mvMBDs.

Subsequent Gene Set Enrichment Analysis (GSEA) of the identified core shared genes revealed pronounced pleiotropic signatures. These target genes were not only highly enriched in classical pathways regulating bone homeostasis and metabolism (involving genes such as *WNT16*, *RSPO3*, and *MEPE*), but also demonstrated overwhelming enrichment in systemic lipid and cholesterol metabolism (e.g., lipoprotein remodeling and clearance, involving *LPL*, *CETP*, and *APOE*), as well as systemic cardiometabolic pathways, including metabolic syndrome and type 2 diabetes (T2D). Additionally, we observed a striking overlap of genetic pathways between these genes and neurodegenerative disorders, particularly Alzheimer’s disease and its associated biomarkers.

This cross-system enrichment network robustly substantiates our core hypothesis: the progression of mvMBDs is not merely a localized mechanical skeletal degeneration, but rather a complex systemic syndrome driven collectively by systemic glycolipid metabolic dysregulation, lipotoxicity, and a deep degenerative network [44].

To further map the specific genomic elements underlying this complex systemic syndrome, our in-depth analysis of the genome-wide partitioned heritability revealed that the vast majority of genetic risk loci associated with mvMBDs are localized within non-coding regions. While sLDSC identifies statistical heritability enrichment rather than direct causality, these enriched non-coding regions are hypothesized to harbor cis-regulatory elements that potentially influence biological processes by modulating proximal target genes. Empirically, the genetic heritability of our investigated phenotypes was exceptionally enriched in histone modification marks indicative of active chromatin states (e.g., H3K4me1, H3K27ac, and H3K9ac, all exhibiting enrichment p < 0.01). This aligns flawlessly with the seminal paradigm established by Finucane et al. (2015) [28], which posits that risk variants for complex genetic traits are disproportionately concentrated within the functional regulatory elements of non-coding DNA [28]. The significant heritability enrichment observed in these epigenetic annotations suggests their statistical importance in capturing disease-associated polygenic variation that may influence individual susceptibility. Further deconstruction of the underlying genetic risk architecture confirmed several pivotal higher-order genomic regulatory elements based on our empirical data. Notably, super-enhancers (Enrichment p = 2.41 × 10^-7^), transcription start sites (TSS), and transcription factor binding sites (TFBS) exhibit profound statistical heritability enrichment [45]. These specific genomic elements capture a substantial proportion of the genetic liability associated with bone microarchitectural and glycolipid metabolic traits. Remarkably, for these complex MBDs, we not only validated the effects of classical promoter and enhancer regions but also observed exceptionally strong heritability enrichment within intronic regions (Intron_UCSC, p = 1.44 × 10^-6^). This critical finding underscores that the vast non-coding intronic transcribed regions not only harbor dense distal regulatory networks but also highly likely contribute to the onset and progression of the metabolic bone disease network (e.g., OP, T2D, and CKD) by regulating alternative splicing or mediating post-transcriptional modification mechanisms [46].

While this investigation provides an integrative framework and valuable insights into the shared genetic architecture of mvMBDs, several limitations warrant acknowledgment. First, the analytical cohorts consisted predominantly of individuals of European descent, which limits the generalizability of our findings across diverse genetic backgrounds. Consequently, future investigations must prioritize the inclusion of multi-ethnic and trans-ancestral cohorts to rigorously validate these genetic discoveries and ensure their broad applicability. Second, the factor loadings within our Genomic SEM framework were heterogeneous across constituent traits, with metabolic and inflammatory markers exerting stronger influences on the latent construct than isolated skeletal phenotypes. Furthermore, the univariate GWAS for CKD possessed a relatively modest heritability Z-score (h^2^ Z = 1.26). Although the model demonstrated robust overall fit indices, these differential loadings suggest that the derived latent factor reflects a broad metabolic-inflammatory disease nexus influencing skeletal biology, rather than an isolated bone pathology. Future investigations leveraging higher-powered univariate GWAS will be valuable to evaluate more granular multi-factor configurations. Third, although fine-mapping and transcriptomic analyses successfully prioritized multiple susceptibility loci associated with mvMBDs, elucidating the precise molecular mechanisms bridging these candidate genes to functional biological pathways remains a challenge. Subsequent experimental studies are warranted to decipher how these genetic variants influence the individual mvMBD phenotype by modulating target gene expression, bone remodeling dynamics, and systemic metabolic networks. Furthermore, while our study highlights the significant contribution of genetic factors to the etiology of mvMBDs, the concurrent influence of environmental determinants cannot be overlooked. Future research endeavors should prospectively investigate how modifiable environmental exposures—such as tobacco use, alcohol consumption, and psychosocial factors like loneliness—interact with genetic predispositions to alter phenotypic expression, thereby advancing our systemic understanding of gene-environment (G × E) interactions in the pathogenesis of mvMBDs.

## Conclusion

In conclusion, the present study provides valuable insights into the shared genetic architecture underlying mvMBDs. By seamlessly integrating Genomic SEM, statistical fine-mapping, and transcriptomic profiling, we successfully characterized multiple shared genetic susceptibility loci not captured by individual single-trait GWAS. Furthermore, we elucidated how these specific variants orchestrate the intricate genetic nexus between targeted gene expression and complex systemic traits. Our findings transcend traditional mechanistic paradigms of MBDs, offering transformative perspectives that will critically inform future precision medicine strategies and targeted public health interventions. Future research endeavors are essential to functionally validate these putative genetic markers and to comprehensively delineate the role of gene-environment (G × E) interactions in the pathogenesis of mvMBDs, ultimately aspiring to extend the global healthspan.

## Supporting information

S1 File Empirical genetic covariance matrix among the six analyzed trai ts. This file contains the empirical genetic covariance and correlation matrix generated via the multivariate extension of cross-trait LDSC. This matrix serves as the statistical foundation and primary input for the subsequent common factor Genomic SEM analysis.
(ZIP)

S2 File Regional association plots for the 59 independent genomic risk loci. This compressed archive contains the comprehensive graphical visualizations and regional association plots for each of the 59 independent genomic risk loci identified via the FUMA pipeline. Each image provides detailed functional annotations and LD structures for the prioritized variants within the respective locus.
(7Z)

S1 Table Model fit evaluation metrics and parameters for the single-factor structural equation model. This table presents the standardized stability evaluation indices used to assess the model fit, including the SRMR, model χ2, AIC, and CFI. Additionally, it details the specific estimated parameters for the latent factor (F1) and the constituent univariate structural equation models.
(XLSX)

S2 Table Detailed single-factor genetic parameters across the analyzed phenotypes. This table provides the comprehensive single-factor genetic parameters and quantitative estimates derived from cross-trait LDSC. It details the strong, pervasive genetic correlations observed among the diverse phenotypic traits evaluated in this study.
(XLSX)

S3 Table Highly significant genome-wide risk loci (p < 5 × 10^-16^). This table details the 156 highly significant genomic loci identified through the indirectly measured multivariate GWAS for mvMBDs.
(XLSX)

S4 Table Details of the 193 lead SNPs not genome-wide significant in constituent single-trait GWAS. This table presents the 193 loci identified among the 197 lead SNPs that did not achieve genome-wide significance in the six constituent univariate GWAS used to construct mvMBDs.
(XLSX)

S5 Table Lead SNPs previously reported in the literature. This table provides the comprehensive identification details for the four highly significant lead SNPs (p < 5 × 10^-16^) that have been previously identified in existing literature.
(XLSX)

S6 Table Independent genomic risk loci identified by FUMA. This table details the 59 independent genomic risk loci identified through the evaluation of Genomic SEM summary statistics using the FUMA pipeline.
(XLSX)

S7 Table Functional annotations of the 70 distinct lead SNPs. This table provides the detailed FUMA-driven functional annotations for the 70 lead SNPs, including their localization to specific genomic regions.
(XLSX)

S8 Table Candidate variants prioritized via the subtraction filtering approach. This table details key candidate SNPs, notably rs10280461 and rs9594738, identified through the subtraction filtering approach.
(XLSX)

S9 Table Detailed results of the mvMBDs fine-mapping and 95% credible sets. This table provides the comprehensive fine-mapping results, detailing the specific linked variants that comprise the 95% credible sets for the complex genomic regions associated with mvMBDs.
(XLSX)

S10 Table Significant genes identified via the TWAS. This table details the 12 genes that successfully surpassed the rigorous multiple-testing correction threshold in the TWAS FUSION pipeline, representing significant gene-level associations underlying the genetic architecture of mvMBDs.
(XLSX)

S11 Table Candidate genes prioritized by TWAS and FOCUS analyses. This table provides detailed statistics for the consensus candidate genes prioritized through TWAS and FOCUS fine-mapping, highlighting the effect directions of genetically predicted expression (positive vs. negative Z-scores) associated with mvMBD genetic liability.
(XLSX)

S12 Table Candidate genes identified via MAGMA gene mapping. This table details the 27 distinct candidate genes successfully prioritized through the MAGMA framework.
(XLSX)

S13 Table GSEA results. This table summarizes the results of the comprehensive gene-set analyses, detailing the highly significant, cross-system pleiotropic enrichment terms identified using the prioritized core genes.
(XLSX)

S14 Table Cell-type-specific enrichment analysis results. This table details the findings from the cell-type-specific enrichment analyses of diverse single-cell profiles, specifically highlighting the significant cellular etiologies (e.g., pancreatic A cells and tracheal mesenchymal cells) that successfully surpassed the robust multiple-testing correction threshold (p < 0.05).
(XLSX)

S15 Table Genetic contribution and heritability enrichment across distinct genomic regions. This table details the impact of genetic contributions across various functional genomic categories, providing evidence that the pathogenesis of the complex comorbid phenotype relies profoundly upon the systemic remodeling of the underlying genomic regulatory landscape rather than being primarily attributable to protein-altering coding mutations.
(XLSX)
